# Small RNA populations reflect the complex dialogue established between heterograft partners in grapevine

**DOI:** 10.1093/hr/uhab067

**Published:** 2022-01-20

**Authors:** Bernadette Rubio, Linda Stammitti, Sarah Jane Cookson, Emeline Teyssier, Philippe Gallusci

**Affiliations:** EGFV, University Bordeaux, Bordeaux Sciences Agro, INRAE, ISVV, F-33882, Villenave d’Ornon, France

## Abstract

Grafting is an ancient method that has been intensively used for the clonal propagation of vegetables and woody trees. Despite its importance in agriculture the physiological and molecular mechanisms underlying phenotypic changes of plants following grafting are still poorly understood. In the present study, we analyse the populations of small RNAs in homo and heterografts and take advantage of the sequence differences in the genomes of heterograft partners to analyse the possible exchange of small RNAs. We demonstrate that the type of grafting per se dramatically influences the small RNA populations independently of genotypes but also show genotype specific effects. In addition, we demonstrate that bilateral exchanges of small RNAs, mainly short interfering RNAs, may occur in heterograft with the preferential transfer of small RNAs from the scion to the rootstock. Altogether, the results suggest that small RNAs may have an important role in the phenotype modifications observed in heterografts.

## Introduction

Grafting has been widely used for hundreds of years for the clonal propagation of vegetables and of woody fruit trees [1]. It consists of the union between two plant segments: a shoot segment, the “scion”, is grafted on a root segment, the “rootstock”, with the aim to generate chimeric plants combining the characteristics of both plant segments. Grafting was initially used to improve the agronomic performance of crops for example, fruit quality in fruit crops such as watermelon and fruit trees like sweet cherry, apple or citrus [1]. More recently, grafting was also shown to enhance abiotic stress tolerance as in cherry tomatoes grafted on drought-tolerant rootstocks or in cucumbers submitted to salt stress. However, a major interest of grafting is the potential of this technique to improve the resistance of plants to pests and diseases [2]. An important example is the successful use of grafting in viticulture to manage phylloxera infection, an insect-pest that destroyed European vineyards after its introduction from America in the middle of the 19^th^ century [3]. In addition to phylloxera, rootstocks contribute to the control of other soil-borne pests and to the response to various abiotic constraints such as drought, salinity, limestone and mineral nutrition problems [4].

Grafting can be performed using plant parts with the same genotype coming either from the same individual (autograft) or from two different individuals of the same genotype (homograft). In contrast, heterografts use plant parts from two different genotypes. Several steps are necessary for a successful grafting which includes the formation of a callus and the differentiation of vascular tissues [5]. The reconnection of xylem and phloem allows bidirectional exchanges between scion and rootstock such as the exchange of water, nutrients, hormones, metabolites, peptides, small organic molecules and nucleic acids [6, 7].

Among these exchanges, small RNA movements between the graft partners have recently been used as a strategy to target gene silencing and to control various physiological processes related to plant development and stress responses [8–10]. Different classes of small RNAs have been identified in plants that are classified in two main groups, the micro RNA (miRNA) and the small interfering RNA (siRNA). In spite of similarity in size (20–24 nucleotides), miRNA differs markedly from siRNA in their synthesis, precursor structures and mechanisms of action [9–11]. Plant miRNAs are encoded by endogenous genes transcribed by RNA Polymerase II into long primary miRNAs (pri-miRNAs) that fold into hairpin-like structures. Pri-miRNAs are cleaved by RNAse II-like Dicer 1 (DCL1) into smaller stem-loop structures called precursor miRNAs (pre-miRNAs) which are processed again by DCL1 to produce the mature miRNA duplexes. The miRNAs mediate Post Transcriptional Gene Silencing (PTGS) through mRNA cleavage or repression of translation [11]. Two main types of siRNAs, deriving from long dsRNAs precursors, have been described depending on their mode of action. The 21/22-nt siRNAs are produced by DCL2 or DCL4 activity from specific genes called *TAS* genes. They most often induce degradation of their target mRNAs through PTGS even though they can also trigger Transcriptional Gene Silencing (TGS) at some specific loci [12]. In contrast, 24-nt siRNAs are generated from heterochromatic regions through successive actions of RNA polymerase IV, RNA-dependent RNA polymerase RDRD2 and DLC3 [12]. They are involved in TGS by guiding the Domain rearranged DNA methyltransferase (DRM) and therefore play an essential role in the RNA-directed DNA methylation (RdDM) pathway [12].

Small RNAs (smRNAs) can move either from cell-to-cell or in a systemic way. Cell-to-cell movement occurs through plasmodesmata which establish cytosolic continuity between adjacent cells whereas the long distance trafficking takes place through the plant vascular system [8, 13–15]. Some studies show that there could be a preferential association of 21 nt smRNAs and 24 nt smRNAs with local and systemic movement respectively [16, 17]. One general assumption to explain the fact that miRNAs appear to be less mobile than siRNA is that 21-nt smRNAs mediate RNA silencing over short distances while 24-nt sRNAs are effectors of silencing over long distance [14]. However, the selection of mobile smRNAs could simply be linked to the quantity of smRNAs within the source compartment which, if too low, does not allow its efficient propagation. Other hypotheses concerning the selectivity of mobile smRNAs linked to biological triggers, other than sRNA size, like biogenesis pathways were suggested [14].

The mobility of smRNAs was first evidenced following their identification in the phloem sap of several plants. For example, 161 miRNAs were detected by microarray hybridization in *Brassica napus* phloem sap [18], and more than 1000 siRNAs in the one of pumpkin [19]. The phloem sap is an RNAse-free environment so the small RNAs could be stable in an unbound form [13]. Moreover, phloem saps of pumpkin, cucumber and lupin contain proteins that have the ability to bind RNA and may therefore protect mobile RNA molecules from degradation [19]. Besides, in pumpkin phloem sap the CmPSR1 (*Cucurbita maxima* Phloem Small RNA Binding Protein1) protein binds selectively to small single-stranded RNAs [19]. However, the detection of small RNAs in the phloem sap is challenging and limited to a few plant species from which the phloem can be extracted in sufficient quantities. Thus, the evidence of small RNA mobility in plants have mainly been studied using grafting, in which part of a plant undergoing RNA-mediated gene silencing is grafted onto a plant that does not experience silencing. Ruiz-Medrano et al. obtained the first evidence of transport of pumpkin mRNA in cucumber scion tissues through heterografting between pumpkin (rootstock) and cucumber (scion) [20]. Both transgene-specific and endogenous siRNAs showed mobility in graft combinations of *Arabidopsis* [17]. Studies on gene silencing established that mobile 24 nt-siRNA from *Arabidospis* rootstock induces gene silencing in the mutant scion that is compromised in 24 nt small RNAs production [13]. Mobility is not limited to source-to-sink movement through the phloem; in *Nicotiana benthamiana* siRNA signals are transported over long distances from shoot-to-root and root-to-shoot in graft combinations using transgenic scion or rootstock [21]. A recent study on sweet cherry trees using scion and rootstock of two very distinct species demonstrated endogenous transfers of small RNAs through small RNA sequencing data analysis [22].

The exchanges of smRNAs across the graft junction can impact the scion and the rootstock physiology and development. The miRNA transmitted over long distances have been shown to be involved in the response to abiotic stress [23, 24]. Epigenetic modifications through mobile small RNAs could create heritable phenotypic variations via grafting [2, 25]. Thus, mobile small interfering RNA from the shoots have been found to direct transcriptional gene silencing in the rootstock cells of *Arabidopsis* [13]. Other examples on how the long distance exchanges of these molecules and signaling factors regulate scion/rootstock relations are reviewed in [6, 8, 14, 15].

Grapevine (*Vitis vinifera*) is a major fruit crop worldwide whose development and productivity are strongly affected by the choice of the rootstock [26]. Transcriptomic studies have shown that the rootstocks can modify scion gene expression [26–28]. Grafting has also been shown to affect miRNA abundance with variations between hetero- and autografted grapevines [23]. All these studies reveal the impact of the rootstock on scion gene expression and miRNAs abundance suggesting that some smRNAs can move across the graft union and could be responsible for changes in scion phenotypes. Therefore, high throughput genome sequencing allows the identification of more than 3000 genes transporting mRNAs across grapevine graft junctions [29].

In the present study, we investigated the smRNA populations in the homograft of *V. vinifera* cv. “Cabernet Sauvignon” (CS) and in two heterografts CS grafted on the commercial rootstock *Vitis riparia* cv “Gloire de Montpellier” (RGM) or onto the *Vitis berlandieri x Vitis rupestris* hybrid cv. 1103 Paulsen (1103P). We show that both homo- and hetero-grafting and the rootstock genotype influences the smRNA populations found in the different parts of grafted plants. Making use of the presence of Single nucleotide polymorphisms (SNP) between the partners of heterografts, we also identify putative smRNA exchanges between CS grafted on the commercial rootstock RGM. Part of the smRNA populations found in either the rootstock or the scion of the heterograft can only be explained by the mobility of smRNA between the graft partners. This exchange of smRNAs between the graft partners may therefore contribute to the reciprocal influence that graft partners have over each other.

## Material and method

### Plant materials and growth conditions

**Figure 1:**
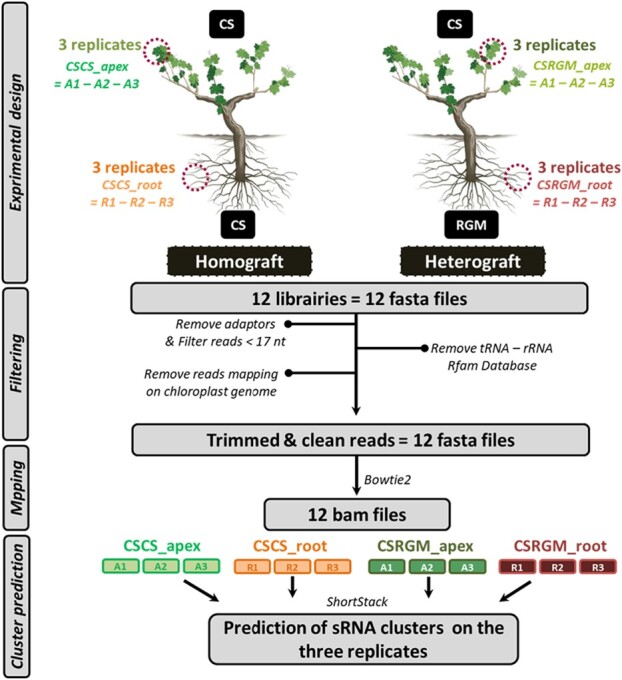
Small RNA analysis strategy. Three replicates for each apex and root samples of two graft combinations were used to prepare a total of 12 small RNA libraries. Sequences were cleaned of adaptors and filtered for a minimal read size of 17 nucleotides. Reads associated to tRNA and rRNA or homologous to the chloroplastic genome were removed after mapping on Rfam databases. Cleaned fasta files were mapped on the CS or RGM genomes depending on the genotypes of the plant tissues analyzed (see methods). ShortStack algorithm was used to predict sRNA clusters with the three replicates of each condition merged.

Scions of *V. vinifera* cv. Cabernet Sauvignon (CS) were either homo-grafted (CS onto CS) or hetero-grafted onto *Vitis riparia* cv. Gloire de Montpellier (RGM) or onto the *V. berlandieri x Vitis rupestris* hybrid cv. 1103 Paulsen (1103P) as described in [30]. After callusing and rooting, grafted plants were cultivated in 3 L sand-filled pots in greenhouse conditions and irrigated with a full nutrient solution as described in [31]. The plants were harvested approximately 4 months after grafting. Shoot (shoot apices and young leaves) and root (tips) samples were harvested from three independent plants per scion/rootstock combination and immediately frozen in liquid nitrogen. In total 18 samples were analyzed (Fig. 1).

### Small RNA library construction and sequencing

Total RNAs were isolated from the root and leaf samples using the Norgen Plant/Fungi Total RNA Purification Kit (BioTek Corp.) with some modifications to the first step of the extraction: 800 μL of lysis buffer was used per sample and the resulting solution was extracted with 800 μL of chloroform:isoamyl alcohol (24:1, v/v) once; the supernatant was used for the subsequent steps in the protocol according to the manufacturer’s instructions. Total RNAs sent to the GeT-Biopuces platform (Toulouse, France). The libraries were sequenced on Illumina Hiseq 2500 as single-end 50 base reads according to Illumina’s instructions. Image analysis and base calling were performed using RTA 1.17.21.3 and CASAVA. The data have been deposited in the National Center for Biotechnology Information (NCBI) Sequence Read Archive (https://www.ncbi.nlm.nih.gov/sra) under accession number PRJNA734864.

### Small RNA-seq data processing

Data analysis was performed on root and apex samples from the homograft CS/CS and the heterograft CS/RGM. Regarding the heterograft CS/1103P, only apex samples were analyzed due to the absence of a sequenced genome of the rootstock 1103 Paulsen. Thus, data related to the CS/1103P heterograft were only used when comparing the scion’s small RNA populations between homograft and heterografts. The quality assessment of raw data was done with FastQC (version v0.11.7) (http://www.bioinformatics.babraham.ac.uk/projects/fastqc). Adaptor sequences were trimmed and reads longer than 17 nucleotides (nts) were retained using TrimGalore! (version v0.4.5) (https://www.bioinformatics.babraham.ac.uk/projects/trim_galore/). Low complexity sequences were removed with the Prinseq tool (version 0.20.4) [32]. To filter tRNA/rRNA sequences and chloroplastic sequences, reads were mapped to the Rfam database sequences and to the grapevine chloroplast genome [33] using Bowtie2 (version v2–2.3.3.1) with no mismatch allowed [34, 35]. Cleaned reads were finally mapped to the RGM or CS genomes using Bowtie2 (version v2–2.3.3.1), depending on the genome of the plant part analyzed [34]. ShortStack (version v3.8.5) was used to identify and quantify small RNA clusters, defined as uninterrupted linear genomic regions, with default settings for all the options (—dicermin 20 —dicermax 24 —mincov 5 raw reads) [36]. Small RNA clusters are built by merging genomic locations, called “island”, covered by reads with a minimum sequencing coverage of 5 reads (mincov option) when these islands are no more distant than 100 nucleotides (pad parameter). The min coverage value was chosen to limit background and increase the specificity of Island detection. As clusters could be composed of a mixture of small RNAs of different sizes, a dicer call score was established: clusters containing at least 80% of reads with a size ranging from 20 to 24-nt were considered as dicer-derived, whereas all others were not and therefore annotated as not dicer-derived and excluded from the following analyses. In addition, clusters were classified in 20 to 24-nt clusters based on the size of the predominant reads found in the cluster. Details of size composition of clusters in the different size classes are provided in the Supplementary Table 1. ShortStack also allows annotating miRNA loci based on a strict set of structural and expression-based criteria as explained in [36, 37]. Detailed data processing steps are shown in Fig. 1. To identify robust clusters that do not depend on individual situations, all cluster determination and analyses were performed in triplicate. However, to account for variability between individual grafts that may arise from the history of the plant segments used for grafting and on plant history after grafting, shortstack used an alignment file (bam file) which includes the information of all the three replicates of each graft combinations at the same time. Information related to each replicate is clearly identified which allows distinguishing the specific and common features of replicates (absence/presence of reads and abundance of expressed reads). For the scions of all graft combinations we show that on average 83.7%, 15.7% and 0.63% of clusters are found in three, two and one replicates respectively (Supplementary Table 2). Therefore, only a tiny fraction of these clusters are found in only one replicate, indicating that the data are more representative of the graft combination rather than of individual replicates.

### Detection of mobile smRNAs moving between scion and rootstock

The mobility of smRNAs was studied in CS/RGM grafts making use of the sequence differences between genotypes. Mobility of smRNAs was analyzed in both directions, from scion-to-rootstock and from rootstock-to-scion. Small RNA mobility analysis was based on a two-step procedure with successive alignments on the genome of the donor compartment and then on the genome of the recipient one. To identify scion-to-rootstock mobile smRNAs, the cleaned reads from the rootstock were first mapped to the RGM genome (rootstock genome) in order to select smRNAs having 100% homology with the RGM genome. The unmapped reads (having at least one mismatch with the RGM genome) were then mapped to the CS genome (scion genome) with no mismatch allowed. Reads that did not map on the RGM genome, but were 100% homologous to the CS one were considered as corresponding to mobile smRNAs from the scion (CS) to the rootstock (RGM). The reverse approach was used to identify smRNAs moving from the rootstock-to-scion. We then used shortstack to perform the cluster predictions associated with these mobile smRNAs. Detailed data processing steps are shown in Supplementary Fig. 9.

### Identification of known miRNAs

Known *V. vinifera* miRNAs (vvi-miRNAs) were identified using the sequences of the 163 vvi-miRNA known sequences present in the miRBase22 (http://www.mirbase.org/). Using a fasta file composed of the sequences of all vvi-miRNA miRNA-hairpins, a blast analysis was performed using NCBI_Blast+ (version v2.6.0+) to create a BLAST database (makeblastdb) that was used to blast the sequences of the clusters annotated as miRNA by shortStack in our samples (blastn - task blastn-short -max_target_seqs 1; e-value threshold of 0.05).

### Genomic locations of smRNA clusters

To identify the genomic locations of dicer derived clusters we used the annotation files including the genomic coordinates of the genes and repeats of RGM [38] and CS [39] genomes. The 2 kb promoter regions were defined as the 2 kb upstream of genes. The annotation analyses were performed with BEDTools (version v2.27.1) by taking the coordinates of the clusters and the coordinates of the genes, 2 kb promoter regions and repeats of each genome. The aim was to identify intersections between the clusters coordinates and the genome annotations files (bedtools intersect –wa –wb). If an intersection is found, the cluster was therefore annotated in accordance with the annotation file with which it intersects [40].

### Gene ontology enrichment analysis

The gene ontology enrichment (GO) analyses were performed on clusters associated with a gene (at the gen body level or at the 2 kb promoter region). GO terms associated with each genome were downloaded (Minio et al. 2019; Girollet et al. 2019). The Bioconductor R package topGO was used to determine GO terms significantly enriched in different gene sets [41]. The statistical significance test selected was Fisher’s exact test with a significance level α < 0.05.

### Selection of potential targets in the recipient compartment

To identify CS genomic regions potentially targeted by the 115 clusters corresponding to mobile 24-nt siRNA, blast analyses were performed on the genome of the receptor compartment (i.e. the CS genome). Blasts with an e-value <0.05 were kept with the exception of those annotated to repeats that were excluded. Based on blast information and taking into account the length of the blasted region, the number of mismatches we calculated a pident_final value (final percentage of identity) that represents the final identity level weighted by the percentage of the cluster that is actually blasted (see Supplementary Data 1 for details).

A total of 10 potential targets were chosen on the basis of the cluster length blasted ranging from 9 nt to 132 nt, the number of mismatches ranging from 0 to 8 and on the value of the final pident ranging from 20% to 97.37%. (Supplementary Table 10). The primers corresponding to the regions potentially targeted in CS (scion) by mobile siRNA clusters were designed on BatchPrimer 3 software and are shown in Supplementary Table 3.

### McrBC PCRs

For methylation analysis, DNAs were extracted from apexes of each replicates separately for each of the two graft combinations CS/RGM and CS/CS, using the Nucleospin Plant II kit (Macherey Nagel), quantified at 260 nm and quality control was performed after electrophoresis on a 1% agarose gel. For McrBC- PCR methylation analysis, 500 ng of genomic DNA was digested with McrBC (NEB) for 3 h in a final volume of 50 μl containing 1 x NEBuffer 2, supplemented with 200 ng/μl BSA, 50 U of the McrBC enzyme according to manufacturer instructions with or without 1 mMGTP as a negative control. PCR amplification was performed with 25 ng of genomic DNA with the relevant primers shown in Supplementary Table 3. PCR reactions were performed in a final volume of 30 μl, (1x green Gotaq reaction buffer, 0.2 mM each dNTP, 0.67 μM of forward and reverse primers, 1 U Gotaq G2 DNA polymerase (Promega) for 27 cycles in standard PCR conditions. Semi-quantitative McrBC-PCR analyses were done with the Image Lab 6.1software BIO-RAD. Difference in genomic DNA cluster digestion levels between CS/CSand CS/RGM grafts were assessed using a Student t test.

## Results

### Small RNAs in grapevine apexes and roots of two scion/rootstock combinations show specific accumulation patterns

To characterize the smRNA populations of grapevine plants, total RNAs extracted from apexes and roots of either homografts (CS/CS) or heterografts (CS/RGM) were used to prepare smRNA libraries. A total of 12 libraries were sequenced with an average sequencing depth of 20 M reads and analyzed using the pipeline presented in Fig. 1.

Briefly, after a filtering step including cleaning of reads (adaptors trimming and size filtering), reads corresponding to rRNA and tRNA (40% of total reads) were eliminated using the Rfam database [35] as well as reads complementary to the chloroplast genome [33]. Reads were subsequently mapped on the CS or RGM genome depending on the origin of the libraries with on average 73% to 80% and 53.4 to 63.6% of mapping efficiency for apex and root samples respectively (Supplementary Table 4). The ShortStack package was subsequently used to predict siRNA and miRNA clusters using the merged data of all replicates for each plant tissue (apex and root) of each scion/rootstock combination. The number of clusters identified represents between 57.3 and 67.5% of the total number of reads that were mapped on the CS and the RGM genomes respectively (Table 1). A total of 101 760, 153 870, 113 245 and 92 661 smRNA clusters were identified in apex and in root samples of CS/CS and CS/RGM respectively (Table 1). The higher number of clusters identified in the apex of the heterograft (1.5 to 2 times more than in other conditions) correlates with the lowest average number of reads per cluster. The opposite situation is observed in the root samples of the heterograft, which presents the lowest number of clusters and the highest number of reads per cluster. This contrasted situation results in clear differences in the proportion of the genome covered by smRNAs. The distribution of clusters based on the number of reads classified into seven categories globally shows a higher number of clusters in the apexes of CS/RGM and a lower number of clusters in the roots of CS/RGM whatever the categories (Supplementary Fig. 1). The distribution of clusters identified in CS/CS is similar between root and apex samples except for clusters with a low number of reads (<=5) which are much less abundant in the CS/CS leaves (about 5 times less abundant compared to root samples, Supplementary Fig. 1).

**Table 1:** Description of the cluster prediction analysis of the four conditions

	CSCS_apex	CSRGM_apex	CSCS_roots	CSRGM_roots
Total number of reads	14 643 414	16 582 099	17 427 942	15 827 728
Reads associated to clusters	9 883 252	10 316 251	9 988 301	9 563 750
% of reads associated to clusters	67.5%	62.2%	57.3%	60%
Total number of clusters	101 760	153 870	113 245	92 661
Number of miRNA clusters	102	103	93	87
Number of siRNA clusters	101 658	153 767	113 152	92 574
Average number of reads per cluster	97	67	88	103
Average number of reads per cluster (rpm)	5.85	3.55	3.17	3.67
Average cluster lenght (in bp)	203	193	188	211

Abbreviation: rpm, total number of reads per cluster normalized to reads per million; bp, base paired

The clusters of each condition were separated into siRNA and miRNA clusters, predicted on the basis of smRNA secondary structure [36]. The miRNA clusters were similarly abundant in the different conditions analyzed and therefore cannot account for differences in the total number of clusters found between scion/rootstock combinations (Table 1). Among the miRNA clusters, 77.5% to 81.7% depending on samples correspond to already identified grape miRNAs and 17% to 22.5% to yet unidentified miRNAs (Supplementary Fig. 2). The miRNA clusters were mostly associated with reads of 21-nt (66.6–74.5%) whereas those corresponding to siRNA clusters are mainly associated with reads of 24-nt (87.4–93.3%, Supplementary Fig. 3).

Clusters were also classified based on their genomic location. Between 78% and 88.73% of the clusters were located in gene bodies, 2 kb promoter regions or repeated sequences (tandem repeats and transposons), the remaining being located in intergenic regions (Supplementary Table 5). The number of clusters annotated in gene bodies represents between 6% and 14% of all mapped clusters, those located in 2 kb promoter regions from 2% to 8.2% and at repeated sequences from 33.8% to 60.5% of all mapped clusters. Between 1.2 to 2.7%, 10.3 to 10.5% and 8.7 to 12.4% of the clusters are annotated in both gene body and 2 kb promoter region, gene body and repeated sequence and 2 kb promoter region and repeated sequence respectively (Supplementary Fig. 4).

### Small RNA populations differ between homo- and heterografts

In order to characterize potential homograft *vs.* heterograft and/or rootstock genotype (RGM *vs.* 11303P) effects on the smRNA (miRNAs and siRNAs) populations found in the scion (CS), clusters identified in the scion of CS/CS were compared to those of CS/RGM and CS/1103P. Cluster analysis of the CS/1103P heterograft was performed with the exact same procedure as for the other two graft combinations (Supplementary Table 6). In total 101 760, 153 870 and 127 638 clusters were identified in the scions of the homograft (CS/CS) and of the CS/RGM and CS/1103P heterografts, respectively. To evaluate the variability between replicates and graft combinations a principal component analysis (PCA) was performed taking into account all the reads used to define the cluster (Supplementary Fig. 5). Results indicate some variability between replicates but allow a clear separation along axis 1 (PC1 which explains 31% of the variance) between homograft and heterograft samples. To explain the apparent variability between replicates visualized by the PCA analysis we controlled their distribution between replicates. Most clusters were found on the three replicates (87.7%, 81.1% and 82.3% for CS/CS, CS/RGM and CS/1103P respectively) and on average 16% in at least 2 replicates confirming the robustness of the approach used (Supplementary Table 2). We therefore analyzed the quantitative variations of reads between the different replicates, by calculating the coefficient of variation (CV: ratio between standard deviation and mean multiplied by 100) which is based on the number of reads found in each replicate. Results indicate that about 40% of the clusters in each graft combination have CV higher than 50%which most likely accounts for the variability between replicates (Supplementary Fig. 6). The two heterografts present a larger number of clusters (153 870 for CS/RGM and 127 638 for CS/1103P) compared to the homograft (101760) with an average number of reads per cluster lower than those of the CS/CS homograft (about 1.5 times less, Supplementary Table 7). The distribution of clusters based on the number of reads shows that the increase in the number of clusters observed in the heterografts correlates with an increase in the number of clusters with a low sequencing depth (<= 5 reads, Supplementary Fig. 7).

**Figure 2:**
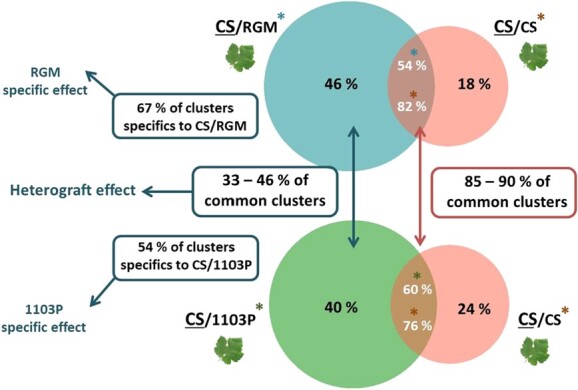
Comparisons of small RNA clusters of the apexes of heterografts vs. homograft. The top Venn diagram corresponds to the comparison of clusters in the apexes of the heterograft CS/RGM vs. the homograft CS/CS. The bottom diagram corresponds to the comparison of clusters in the apexes of the heterograft CS/1103P vs. the homograft CS/CS. The clusters identified in common between heterograft and homograft are referenced by a blue, green and orange star for homograft CS/CS, heterograft CS/RGM and heterograft CS/1103P, respectively. The results of the overlap search between the common clusters of the two Venn diagrams are presented in the red box. The results of the overlap search between the specific clusters to each heterograft scion are presented in the blue box by distinguishing the common clusters and those remain specific to each heterograft.

Qualitative comparisons between samples are based on the presence/absence of smRNA (siRNA and miRNA) and not on their relative abundance. Thus, common clusters correspond to genomic locations where the smRNA clusters of different graft combinations co-localize. Results indicate that most clusters of the homograft are also found in the heterografts (82% and 76% common clusters between CS/CS and CS/RGM and CS/CS and CS/1103P respectively) (Fig. 2). To determine whether common clusters were similarly represented in the different graft combinations, we analyzed their sequencing depth. The number of reads associated with common clusters is in most cases very similar between graft combinations, and in most cases (70.1% - 73%) below 10 reads and (24.2% - 21.9%) between 10 and 50 reads (for details, Supplementary Fig. 8). Furthermore, most of these clusters are common to both comparisons (85% of CS/CS *vs.* CS/RGM and 90% of CS/CS *vs.* CS/1103P suggesting that they correspond either to clusters characteristics of the CS scion and/or are generated by grafting, independently of the type of graft (red arrow and box - Fig. 2). Additionally, 40% (CS/RGM) and 46% (CS/1103P) of smRNA clusters are found in the CS scions of each heterograft and are absent from the CS/CS homograft. Among these clusters, 33% to 46% are common to both heterografts suggesting that they are generated independently of the genotype of the rootstock. In contrast the remaining clusters 67% (CS/RGM) and 54% (CS/1103P) are specific to the scion of each heterograft, consistent with genotype specific effects (blue arrow and box - Fig. 2).

Gene ontology (GO) enrichment analysis performed on the clusters found in the scion of all graft combinations (CS/CS, CS/RGM and CS/1103P) showed that they were enriched in biological functions linked to signal transduction, cell communication, and response to stresses including a large number of disease resistance genes encoding proteins that belong to the NBS-LRR class, ankyrin repeat family protein-like genes and cell wall associated kinases. Similarly, the GO biological process photosynthesis, related to clusters annotated in 2 kb promoter regions, was also enriched. The common clusters between both heterograft scions are enriched in biological processes associated with the regulation of nucleobase-containing compound metabolic processes and translation linked in particular to genes encoding ribosomal proteins. Finally, GO enrichments specific to each heterograft are identified in relation with their specific clusters. Thus, in CS/RGM scions an enrichment in biological functions associated with cellular protein modifications, nucleobase-containing metabolic compound, regulation of gene expression and embryo development while at CS/1103P scions, the biological functions significantly enriched are linked to lipid metabolic process and multicellular organism development (Supplementary Data 2 - Supplementary Table 8).

### Identification of small RNAs exchanges between the scion and the rootstock

In order to identify putative mobile smRNAs between the plant partners in heterografts, we took advantage of SNPs found between the genomes of the rootstock (RGM) and of the scion (CS). A stepwise strategy was developed to analyze smRNA populations present in apexes and roots. Small RNA reads of the receptor tissue were first aligned to the corresponding genome (CS and RGM respectively for the apexes and the roots) and those 100% homologous to this first genome were considered as non-mobile smRNAs. Unmapped reads were subsequently aligned to the genome of the second graft partner (donor tissue) and those that were 100% homologous to this second genome were considered as potentially migrating from the donor to the receptor compartment. Finally, a cluster prediction with shortstack was performed to characterize the mobile smRNA clusters (Supplementary Fig. 9).

**Figure 3:**
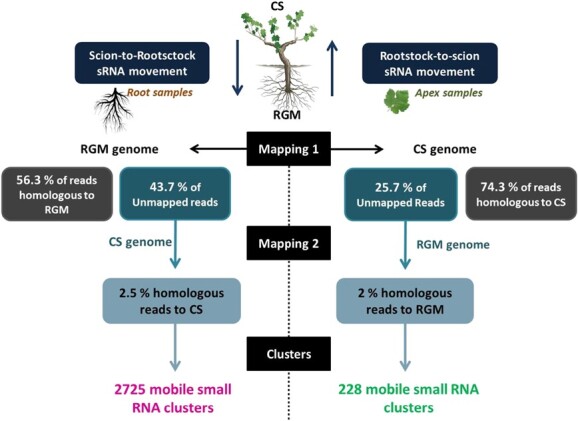
Mobile small RNAs in the heterograft CS/RGM. Scion-to-Rootstock sRNA movement: The analysis is performed on root samples (CSRGM_root, R1-R2-R3). Data were mapped to the RGM genome (Bowtie2–0 mismatch). Unmapped reads were aligned on the CS genome (Bowtie2–0 mismatch) to identify those originating from the scion (CS genome). Cluster prediction was then performed with the ShortStack algorithm. Rootstock-to-Scion sRNA movement: The analysis is performed on leaf samples (CSRGM_apex, A1-A2-A3). Datas were mapped to the CS genome (Bowtie2–0 mismatch). Unmapped reads were aligned on the RGM genome (Bowtie2–0 mismatch) to identify those originating from the roostock (RGM genome). Cluster prediction was then performed with the ShortStack algorithm.

This strategy was used to identify scion-to-rootstock (SC-RT) mobile smRNAs by aligning all reads of the root replicates to the RGM genome, and for rootstock-to-scion (RT-SC) mobile smRNAS by aligning those of the apex replicates to the CS genome. For SC-RT mobile smRNAs, reads that did not map to the RGM genome were subsequently aligned on the CS genome, and the converse for the RT-SC mobile smRNAs. A total number of 15 827 728 reads (56.3% of the total number of reads identified in the three root replicates) were 100% homologous to the RGM genome and therefore considered as non-mobile smRNAs, whereas among the 12 306 348 unmapped reads (43.7% of the total number of reads) a total of 306 570 reads (2.5% of the unmapped reads) were 100% homologous to the CS genome (scion) and therefore considered as SC-RT mobile smRNAs (Fig. 3). For the RT-SC mobile smRNAs, among the 22 332 432 reads identified in the three scion replicates, 16 582 099 reads (74.3% of the total reads) were 100% homologous to the CS genome and 5 750 333 reads (25.7% of the total reads) could not be mapped. Among the latter, 113 221 reads (1.96% of the unmapped reads) were 100% homologous to the RGM genome and therefore considered as RT-SC mobile smRNAs (Fig. 3). We further performed a cluster analysis of these reads as described in the methods and identified 2725 and 228 smRNA clusters considered as SC-RT and RT-SC mobile smRNA clusters respectively (Fig. 3 - Table 2). All mobile smRNA clusters (2725 SC-RT and 228 RT-SC) correspond to siRNA, except one (SC-RT) that was identified as a miRNA cluster (cluster N°2653 – Chr17:6813566–6 813 650). The siRNA clusters were distributed in two main classes, the 21-nt (33.1% and 31.6%) and 24-nt (60.4% and 50.4%) siRNAs for SC-RT and RT-SC mobile clusters respectively (Table 2).

**Table 2:** Characteristics of siRNA clusters identified in movement between scion and rootstock of CS/RGM combination

	Scion-to-Rootstock	Rootstock-to-Scion
Total number of clusters	2725	228
Number of siRNA cluster - 20 nt	38	12
Number of siRNA cluster - 21 nt	901	72
Number of siRNA cluster - 22 nt	86	20
Number of siRNA cluster - 23 nt	54	9
Number of siRNA cluster - 24 nt	1646	115

We then analyzed the distribution of the mobile siRNAs along the genome. Both for SC-RT and RT-SC mobile clusters (Supplementary Fig. 10 and Supplementary Data 3 and 4) 21-nt siRNAs (19.3% and 23.6% of total RT-SC and SC-RT siRNAs, respectively) are associated with gene bodies, whereas 24-nt siRNAs were mostly associated with 2 kb promoter region (6.6% and 10.3% of total RT-SC and SC-RT, respectively) and repeats (23.2% and 38.2% of total RT-SC and SC-RT, respectively) (Supplementary Fig. 10).

When GO enrichment analysis was performed no specific enrichment was identified for clusters associated with 2 kb promoter sequences, irrespective of the direction of the siRNA mobility, in contrast with those associated with gene bodies. In this latter case, processes linked to stress responses, signal transduction, cell communication and lipid metabolism were enriched in putative SC-RS mobile siRNAs (Supplementary Table 9B) and signal transduction, malate transport and superoxide metabolic processes in RS-SC mobile siRNAs (Supplementary Table 9A). Many of the predicted target genes of the mobile siRNAs are involved in stress responses, which is similar to the genes known to be differentially expressed in response to grafting with a non-self partner [24] [26] [30].

### Mobile SmRNAs may trigger DNA methylation in the scion

**Figure 4:**
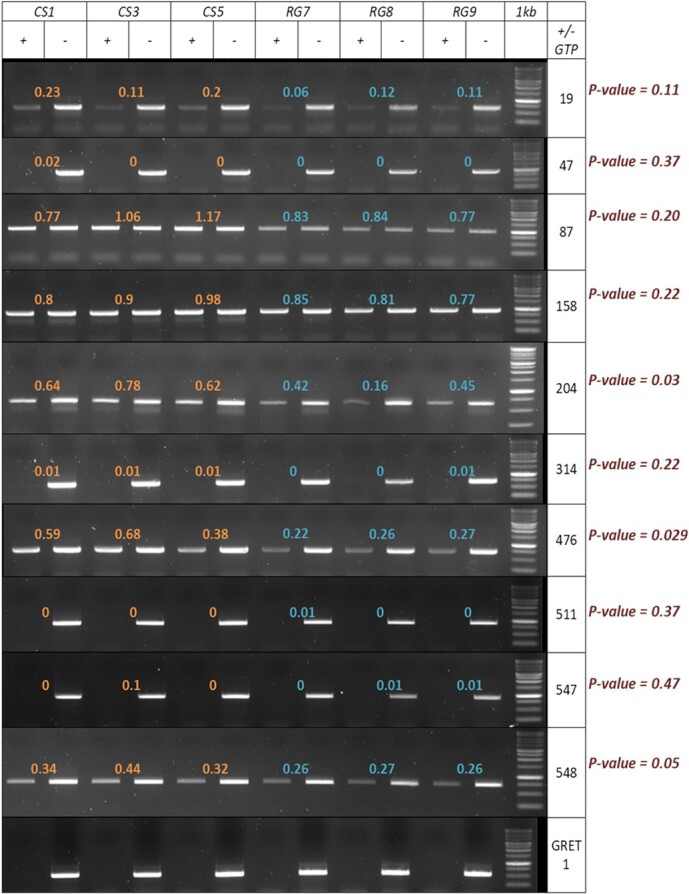
McrBC-PCR analysis of selected loci in apexes of the CS/CS (CS) homograft and RGM/CS (RG) heterograft. 0.5 μg of DNA was digested with McrBC (NEB) during 3 h (+); (−) indicates negative control for the digestion reaction that was performed without GTP. Cluster names are indicated on the right. Plant names and replicate number are indicated (CS1 to 3; RG1 to 3). Ratio between the digested and control band is indicated for each plant and cluster. The P-value of the comparison between the 3 CS versus the 3 RG replicates for each cluster is indicated. Significant differences are found for clusters 204, 476, 548 (P-value = < 0.05) which are more digested in the apexes of RGM/CS plants, indicative of a higher methylation level, whereas all others were similarly digested in the CS/CS and RGM/CS replicates. The GRET1 retrotransposon is used as a control for highly methylated locus, and the 87 and 158 loci as non-methylated DNA.

In order to assess whether mobile smRNAs originating from the RGM rootstock could influence the methylation at the targeted loci we randomly choose 10 different siRNA clusters corresponding to different types of situation in terms of size of cluster and number of mismatches between the smRNA cluster and the CS genome (Supplementary Table 10). Each locus was analyzed using the methylsensitive-PCR (McrBC-PCR) analysis. As shown in Fig. 4, among the 10 selected loci, 3 namely 548, 204 and 476 showed significant methylation differences in the CS apex depending on the RS (Fig. 4).

In all cases methylation was higher with RGM as RS than CS, consistent with the idea that in these cases the mobile smRNA led to an increase in the methylation level in the scion. Interestingly some other loci were either highly methylated in both situations (19, 47, 314, 511, 547), as was the GRET1 retrotransposon [42]used here as a control for highly methylated sequence. In contrast two loci (87, 158) were not digested at all by McrBC showing that they were not methylated irrespective of the RS used. In these cases mobile smRNA seemed not sufficient to trigger methylation in the incipient cells of the scion.

## Discussion

### Identification and characterization of smRNA populations in grafted grapevine combinations

The characterization of smRNA populations was performed on roots and/or apex samples of homografts of *V. vinifera* cv. Cabernet sauvignon (CS/CS) or heterografts using Cabernet sauvignon scion grafted on *V. riparia* cv. Gloire de Montpellier rootstock (CS/RGM) or in *V. berlandieri x V. rupestris* hybrid cv. 1103 Paulsen (1103P) using high-throughput sequencing coupled with robust bioinformatic pipelines that allowed the identification of clusters of smRNAs. In our study, the identification and classification of smRNAs were performed with the Shortstack software [36]. Shortstack allows the characterization of smRNA clusters determined from the mapped reads on the reference genomes showing continuous genomic coordinates. This tool also allows the identification of miRNA clusters based on a strict set of structural and expression-based criteria. SmRNA clusters are also classified on the basis of the predominant small RNA size found on each cluster [36, 37].

Only a very limited number of clusters (< 1%) corresponding to miRNAs were identified in apexes and roots of both graft combinations. Most of these miRNAs clusters (between 77.5 and 81.7%) correspond to already described and annotated grapevine miRNAs (miRBase). The remaining clusters could correspond to specific miRNAs found in the scion/rootstock combinations analyzed.

In addition, most miRNA (between 67% and 75%) are 21-nt long whereas between 87% and 93% of siRNAs clusters correspond to 24-nt siRNAs and 5% to 11% to 21-nt siRNAs consistent with previous results obtained in several plants including Arabidopsis, tomato, rice and maize [36, 43].

### Apex smRNA populations are specific to each graft combination

Comparing the homograft (CS/CS) to the heterografts (CS/RGM and CS/1103P) revealed differences in number and distribution of smRNA clusters. As only CS was used as scion in this study, they cannot be explained by differences in the genotype of the scion. In addition, the accumulation of new siRNAs was observed in both heterografts irrespective of the genotype of the rootstock that was used, RGM or 1103P. This would suggest that the synthesis of new siRNAs are the consequences of a genotype interaction generated by the heterograft situation itself. Thus, we have identified clear differences in the number and characteristics of siRNA clusters depending on the type of graft performed. Heterografts presented a higher average number of clusters in leaves compared to homograft. These differences are mainly due to an increased number of clusters with a low coverage (<= 5 reads) in heterograft apexes (19.7% and 20% of total clusters of CS/RGM and CS/1103P scions respectively) compared to those of the homograft (4% of total clusters), even though equivalent sequencing depth was obtained. As a consequence, clusters identified in apexes of both heterografts target a larger number of genomic regions in comparison to the homograft apexes. Moreover, the comparative analysis of the smRNA profiles between the two heterografts (CS/RGM and CS/1103P) and the homograft (CS/CS) shows that the majority of the clusters found in the homograft (between 76% and 82% of the total number of clusters) are also present in both heterografts. These clusters presumably correspond to smRNA populations constitutively present in CS scions although we cannot exclude that some of them are generated following grafting irrespective of the rootstock used. This would be consistent with the observation that targeted sequences are enriched in genes involved in the response to stress and cellular communication. Enrichment in such biological functions has already been described in transcriptomic analysis of rootstock/scion interactions [27, 28, 44]. The comparisons of the smRNA of the scions also indicate that 33% to 46% of clusters identified in the scions of CS/RGM and CS/1103P are common to both heterografts and may therefore reflect an heterograft effect. However we cannot exclude that the regions targeted by the newly synthesized siRNAs are in part determined by the rootstock as regions covered by these new siRNAs were different in the CS/RGM heterograft compared to the CS/1103P. Indeed, the comparisons of the smRNA of the scions reveal that 67% and 54% of the clusters identified in the scions of CS/RGM and CS/1103P remain specific to each heterograft and may rather correspond to a genotype effect of the rootstock on smRNA populations of the scions. It has been widely demonstrated that different rootstocks confer different yield and phenological characteristics to the grapevine scions [4]. Transcriptomics studies reveal major changes in gene expression especially between homograft and heterograft [27, 28, 44]. Although we have not analyzed in the present work the mRNA populations of the different graft combinations, it is tempting to hypothesize that changes in smRNA populations determined by both genotypes and the type of graft contribute to the changes in mRNA populations observed after grafting. Thus, new high-throughput sequencing-based analysis could be considered in order to analyze possible links between changes in smRNA populations and transcriptional reprogramming.

### HeteroGrafting reveals bilateral exchanges of siRNAs

After the establishment of the graft union, communication between the rootstock and the scion is bi-directional [6, 7, 25]. In order to identify reciprocal smRNAs transfers between scion and rootstock we have developed a bioinformatic strategy based on the sequence differences (SNP) existing between the genomes of the partners used in the heterograft. The use of heterografts provides an efficient way to identify the origin of mobile RNAs if the genomes of partners present sufficient differences in their sequences. For example, exchange of mRNA in grapevine heterografts was demonstrated using diagnostic SNP to distinguish between scions and rootstocks [29]. In the sweet cherry tree, the mobility of endogenous smRNAs was determined by making use of differences in ploidy levels between the scion and rootstock genomes [22]. Indeed, sRNA exchanges are most likely underestimated as only those presenting sequence differences between partners can be identified.

We have identified bidirectional exchanges of smRNA and shown that clusters of siRNAs migrating from scion-to-rootstock are twelve times more abundant than those migrating from scion-to-rootstock. This is consistent with previous data in heterografts of *Arabidopsis thaliana*, soybean, sweet cherry and common bean also showing a preferential scion-to-rootstock migration most likely due to the source-sink flux [8, 13, 17, 22, 45]. All mobile clusters, except one which correspond to an already characterized miRNA [46, 47] are siRNAs. This result supports the assumption associating short range mobility to miRNAs and long range mobility to siRNAs [15].

### Potential functions of small RNA movement

When clusters of mobile siRNAs were sorted according to their size, most of them corresponded to 24-nt siRNAs (>50%) followed by 21-nt siRNAs (~30%) giving an average 21-nt/24-nt ratio of 55%. This differs significantly from the distribution of the siRNA clusters characterized in leaves and roots of the CS/RGM combination where the 21-nt/24-nt ratio is 7%, suggesting that the migration of siRNA does not only depend on their concentration in the donor tissues, but may be selectively controlled. Selectivity in siRNAs mobility has also been suggested in *Arabidopsis* as only one third of the 24-nt siRNAs are mobile in this plant [17, 48], and siRNAs movement from the pollen vegetative cell into the sperm cells only concerns the 21 nt class [15]. Despite these two examples, the question of the mobility of smRNAs in terms of selectivity and mechanisms of transport remains largely unexplored. For example, it is not clear whether it is the mature forms or the precursors of the mature smRNAs that are transported as both were identified in phloem saps [18, 49]. If smRNA are transported as duplexes, therefore they could act directly in the receiving compartment after being loaded into AGO protein complexes [49]. However, it is very likely that mobile sRNAs do not act directly in the receiving compartiment, but first need to be amplified by the RNA Polymerase IV and/or the RNA-dependent RNA polymerase 6 (RDR6). In this case, the single-stranded sRNA is used as a template for the synthesis of a double-stranded smRNA converted to smRNAs by the action of DCL (mainly DCL4 and DCL2) [8, 13, 50].

The genomic annotations of 21-nt clusters corresponding to mobile siRNA show that they are essentially located within the body of genes, and could influence the translation or stability of the corresponding mRNAs. In contrast, 24-nt clusters are essentially associated with 2 kb promoter regions and repeats, and could mediate transgressive methylation in the incipient cells. Indeed 24-nt siRNAs have been associated with RdDM and TGS, whereas the primary function of 21-nt siRNA seems to be associated with PTGS [10, 11]. The prevalence of 24-nt clusters would support the assumptions that long-distance transport of smRNAs is preferentially associated with those of 24-nt and may result in TGS [48, 51].

Previous studies using a two component transgene system had allowed demonstrating that TGS could spread through the graft junction in a bidirectional manner although the process was much more efficient in the scion to RS direction than the reverse (reviewed in [52]). Additional evidence of the involvement of mobile siRNA in transgressive DNA methylation in heterografts was also reported in the model plant *Arabidopsis* when siRNAs produced from the scion were shown to generate methylation at normally un-methylated loci of the rootstock [48]. Similarly, endogenous repeated sequences located in the scion were shown to induce DNA methylation in the rootstock by generating mobile siRNAs of 24nts (reviewed in [52]). As far as perennials are concerned, although DNA methylation reprogramming has been evidenced after heterograft of rubber tree [53] and citrus under drought stress [54], direct evidence that these methylation changes are associated with mobile siRNA is missing. Similarly changes in DNA methylation associated with heterograft were recently reported in citrus plants using a genome wide approach. In this later case the DNA methylation level together with the abundance of siRNA of 24-nt was reduced in the scion. Hence it is unclear whether these changes are direct effects of mobile 24-nt siRNA or are indirect consequences of genome interaction in the heterograft [55].

Here we provide evidence that loci targeted by mobile siRNA are more methylated in the heterograft RGM/CS combination than in the CS/CS homograft. Of course, only a few of the targeted loci were shown to present an increased methylation level. However, although highly sensitive the McrBC methods require amplifying fragments that are larger than the targeted locus, which may lead to an underestimation of the differential methylation between the CS/CS and RGM/CS grafts. Despite these limitations 3 out of the 10 loci tested displayed differential methylation levels consistent with the idea that heterografting may lead to transgressive methylation mediated by mobile siRNAs in grapevine plants.

## Conclusion

Through the analysis of small-RNA populations in apexes and roots of grafted combinations of grapevine, we have developed a bioinformatic approach based on the sequence differences between the genomes of scion and rootstock to demonstrate a bi-directionnal small RNA transfers between graft partners that may lead to changes in DNA methylation profiles. Understanding the functions of mobile smRNAs will now require evaluating their impacts on mRNAs populations and DNA methylation landscape, in relation with plant phenotypes.

## Acknowledgments

The authors acknowledge Cyril Hévin, Jean-Paul Robert, Jean-Pierre Petit and Bernard Douens for grafting and growing the plants, and Virginie Lauvergeat for sample harvesting. We thank the INRA Genotoul bioinformatics platform (http://genotoul.toulouse.inra.fr) for providing computational resources. Sequencing was performed at the GeT-Biopuces platform (Toulouse, France). This work was supported by grants from the Conseil Interprofessionnel du Vin de Bordeaux (contract number 28325) and France-AgriMer. BR was in receipt of a grant from the “Région Nouvelle Aquitaine”(contract number 22001128 EPISTORE) and from the Labex Cote (METDRY).

## Author Contributions

BR and PG designed the analytical pipeline, performed the data analysis and wrote the manuscript. SC contributed to the experimental design, field trial experiments and manuscript modification. ET and LS performed molecular analysis and contributed to manuscript modification. All authors helped in the revision of the manuscript. All authors read and approved the final manuscript.

## Data availability statement.

The data have been deposited in the National Center for Biotechnology Information (NCBI) Sequence Read Archive (https://www.ncbi.nlm.nih.gov/sra) and are available under the accession number PRJNA734864.

## Conflict of interest statement

Authors declare no conflict of interest

## Supplementary data

Supplementary data is available at *Horticulture Research Journal* online.

## Supplementary Material

Web_Material_uhab067
