# Supplementary material for: Small RNA populations reflect the complex dialogue established between heterograft partners in grapevine
Source: Hortic Res. 2022 Jan 20;9:uhab067. doi: 10.1093/hr/uhab067 (PMC8935936; doi:10.1093/hr/uhab067)
Supplement: Web_Material_uhab067 [file web_material_uhab067.zip › Description of supplementalinformation content.docx]

**Description of Additional Supplementary Files**

**File name :** Supplementary Data 1

**Description :** Results of the identification of potential targets in the recipient compartement (CS scion of the CS/RGM heterograft) of the 115 siRNA clusters of 24-nt migrating from rootstock to scion. After blast analysis of the 115 siRNA clusters sequences on the CS genome, only the results with a p-value < 0.05 were kept. Based on blast information and taking into account the length of the blasted region, the number of mismatches we calculated a pident_final value (final percentage of identity) that represents the final identity level weighted by the percentage of the cluster that is actually blasted. The different steps necessary for the calculation of the pident_final value are the following : Thus i) calculation of the percentage of clusters really blasted (% cluster blast) ii) calculation of a percentage of identity (pident_1) which takes into account the number of mismatchs on the blasted length (=100-((number of mismatch*100)/blasted length)  and finally iii) calculation of the final percentage of identity (pident_final) which is weighted by the percentage of the cluster sequence that is actually blasted (=(pident_1*% of the blasted cluster)/100.

**File name :** Supplementary Data 2

**Description** : Gene ontology enrichment results for 1) the common clusters identified in the comparisons between the homograft scion (CS/CS) and both heterograft scions (CS/RGM and CS/1103P) 2) the common clusters identified in the comparisons between the two heterografts (CS/RGM vs. CS/1103P) 3) the specific clusters identified in the scions of the heterograft CS/RGM and 4) the specific clusters identified in the scions of the heterograft CS/1103P.

GO was performed on clusters annotated at gene bodies and at 2 kb promoter regions.

**File name :** Supplementary Data 3

**Description** : Description of annotation of mobile rootstock-to-scion clusters. Only the clusters having a genomic annotation in gene bodies, 2 kb promoter regions and repetas are presented. Each clusters is decribed by it ID number, genomic coordinates and size. The information of the genomic annotation and functionnal annotations are reported.

**File name :** Supplementary Data 4

**Description** : Description of annotation of mobile scion-to-rootstock clusters. Only the clusters having a genomic annotation in gene bodies, 2 kb promoter regions and repetas are presented. Each clusters is decribed by it ID number, genomic coordinates and size. The information of the genomic annotation and functionnal annotations are reported.
