# Supplementary material for: Small RNA populations reflect the complex dialogue established between heterograft partners in grapevine
Source: Hortic Res. 2022 Jan 20;9:uhab067. doi: 10.1093/hr/uhab067 (PMC8935936; doi:10.1093/hr/uhab067)

Supplementary information

Supplementary Table 1. Distribution of clusters according to the percentage of the predominant category of size

	[20-30 %]	]30-50 %]	]50-80 %[	>= 80 %	Total
**CSCS_apex**	111	3801	24958	72890	101760
**CSCS_root**	256	5299	18915	88775	113245
**CSRGM_apex**	193	5243	25970	122464	153870
**CSRGM_root**	278	4972	15347	72064	92661
**CS1003P_apex**	115	3158	18296	106069	127638

The different categories correspond to the percentage of reads associated with the size category considered as predominant for each cluster and which allows to determine the cluster size.

Supplementary Table 2. Distribution of clusters in the scions of the three replicates of the three graft combinations

	CS/CS	CS/RGM	CS/1003P
**Total number of clusters**	101760	153870	127638
**% of cluster with reads on the three replicates**	87.7 (89200)	81.1 (124795)	82.3 (105069)
**% of cluster with reads on two replicates**	12 (12245)	18 (27749)	17 (21778)
**% of cluster with reads on only one replicate**	0.3 (316)	0.9 (1327)	0.7 (791)

The total number of clusters in the scions of each graft combination are represented as well as the percentage of clusters identified in i) three replicates ii) in at least two replicates and iii) in a single replicate

Supplementary Table 3. Sequence of primers corresponding to the 10 potential targets

Coordinates of the clusters	Cluster_ID	Forward sequence	Reverse sequence	Amplicon size
CM014908.1:1061180-1061218	Cluster_87	AGCGCAAGAGTGAATTGGAG	TGTGAGCATATGACACCCTTG	1013
CM014904.1:23780989-23781025	Cluster_19	TCATAACAGCACAACGCAGAG	ATTGTCGATGAACGATGTGC	655
CM014906.1:16242012-16242130	Cluster_47	CAATGGGGTCAGGAATTAACA	AACACTCGCACCCAACTGTA	882
CM014919.1:5207038-5207061	Cluster_476	TGAAGXACCAATGCAATCTT	TCACCACCATTCTGTTGTGC	699
CM014910.1:19875438-19875633	Cluster_158	TATGGGATCACCTGCTGGTTTAC	TCAAGGCCTCATTCAAGTGG	902
CM014911.1:18899211-18899306	Cluster_204	TGGCCCTCTATTCACGTTTC	TTGAGTCTTTGGGCTTGAGG	690
CM014914.1:13151822-13151949	Cluster_314	TGGCTCCCACTTGAGCTACT	TTGAGTCTTTGGGCTTGAGG	672
CM014921.1:5180682-5180790	Cluster_548	CTCGTCTGCCGCTTAAACA	TTGCGATTTGACTGACGAAC	724
CM014919.1:22655943-22656048	Cluster_511	GCCACCATCTAAGCACCATC	GTCACAATGGTCTCGCTTAA	896
CM014921.1:5166131-5166266	Cluster_547	TGCACTGGAGTCAGACGAAC	CATAGATGTGGTCCGATCCTA	897

Supplementary Table 4. Metrics of small RNA libraries prepared from the apexes and root of CS/CS and CS/RGM

Sample_ID	Replicates	Total_reads	Reads after Rfam cleaning	% of ME on chlorplastic genome	Reads after chloroplast mapping	Reads mapped on CS (1) or RGM (2) genomes	% of ME
**CSCS_apex (1)**	**A1**	17 138 850	11 917 454	46,6%	6 367 954	5 079 419	80%
	**A2**	17 660 428	11 945 292	46,6%	6 382 215	4 671 556	73,2%
	**A3**	17 007 309	10 888 474	38,7%	6 671 574	4 892 439	73,3%
**CSRGM_apex (1)**	**A1**	21 123 054	14 051 047	49,9%	7 036 896	5 223 031	74,2%
	**A2**	27 270 022	17 138 473	50,7%	8 453 264	6 173 356	73,0%
	**A3**	17 935 824	11 569 538	40,9%	6 842 152	5 185 712	75,8%
**CSCS_roots (1)**	**R1**	19 000 776	10 778 009	1,69%	10 596 028	5 657 402	53,4%
	**R2**	21 913 433	10 920 018	2,02%	10 699 154	6 799 682	63,6%
	**R3**	16 367 994	8 682 370	1,96%	8 511 889	4 970 858	58,4%
**CSRGM_roots (2)**	**R1**	17 352 754	9 301 603	2,07%	9 108 835	4 941 666	54,3%
	**R2**	17 002 799	9 212 916	1.87%	9 040 189	5 081 687	56,2%
	**R3**	19 077 555	10 174 221	1.89%	9 982 377	5 804 375	58,1%

ME : Mapping Efficiency

Supplementary Table 5. Clusters annotated on the total number of clusters

Clusters annotated correspond to clusters located at gene bodies, in 2 kb promoter regions or in repeated sequences (tandem repeats and transposons)

	CSCS_apex	CSRGM_apex	CSCS_root	CSRGM_root
**Total clusters**	101 760	153 870	113 245	92 661
**Total of clusters annotated**	90532	136707	99727	71501
**% of clusters annotated**	88.96 %	88.85 %	88%	77.69 %

Supplementary Table 6. Metrics of small RNA libraries prepared from the apexes of CS/1103P

Sample_ID	Replicates	Total_reads	Reads after Rfam cleaning	% of ME on chlorplastic genome	Reads after chloroplast mapping	Reads mapped on CS	% of ME
CS1103P_apex	A1	16 234 077	9 683 689	47.2%	5 110 340	3 695 297	72%
	A2	16 799 640	9 659 000	38.2%	5 974 184	4 543 051	76%
	A3	21 249 211	14 023 915	51.9%	6 744 911	4 593 927	68%

ME : Mapping Efficiency

Supplementary Table 7. Characteristics of smRNA clusters identified in the scions of the homograft (CS/CS) and the heterografts (CS/RGM & CS/1103P)

	CSCS_apex	CSRGM_apex	CS1103P_apex
**Total number of clusters**	101 760	153 870	127 638
**Number of miRNA clusters**	102	103	95
**Number of siRNA clusters**	101 658	153 767	127 543
**Reads associated to clusters**	9 883 252	10 316 251	6 992 432
**Average number of reads per cluster**	97	67	55
**Average cluster lenght (in bp)**	203	193	188

rpm : total number of reads per cluster normalized to reads per million ; bp : base paired

Supplementary Table 8. GO term enrichment analysis of clusters annotated in gene bodies or promoter found in the scions of the homograft (CS/CS) and/or the heterografts (CS/RGM – CS/1103P)

Only the enrichment of the GO related to biological functions are presented. The GO enrichment were done on 1/ clusters found in common between the two comparative analysis CS/CS vs. CS/RGM and CS/CS vs. CS/1103P (‘commons_gene’ and ‘commons_promoter’) 2/ clusters found in common in both heterograft scions (‘commons_heterograft_gene’ and ‘commons_heterograft_promoter’) 3/ clusters specifically found in the scion of CS/RGM (‘specific_RGM_gene’ and ‘specific_RGM_promoter’) and 4/ clusters specifically found in the scion of CS/1103P (‘specific_1103P_gene’ and ‘specific_1103P_promoter’).

Clusters_ID	Go term	Description	Annotated	Significant	Expected	p-value
**Commons_gene**	**GO:0007165**	Signal transduction	5361	570	473.60	5.8e-07
	**GO:0007154**	Cell communication	6422	679	567.33	0.001
	**GO:0006629**	Lipid metabolic process	3920	386	346.30	0.011
	**GO:0040029**	Regulation of gene expression	359	44	31.71	0.017
	**GO:0006950**	Response to stress	8565	806	756.65	0.018
**Commons_promoter**	**GO:0015979**	Photosynthesis	505	97	81.82	0.039
**Commons_heterograft_gene**	**GO:0006139**	Nucleobase containing compound metabolic	13507	129	109.53	0.016
**Commons_heterograft_promoter**	**GO:0006412**	Translation	2025	61	48.05	0.034
		Pollen-pistil interaction	385	15	9.14	0.043
**Specific_RGM_gene**	**GO:0006464**	Cellular protein modification process	8967	552	455.09	1.1e-07
	**GO:0006139**	Nucleobase containing compound metabolic	13507	724	685.50	0.0064
	**GO:0040029**	Regulation of gene expression	359	26	18.22	0.00448
	**GO:0009790**	Embryo development	1302	80	66.08	0.0453
**Specific_RGM_promoter**	**GO:0007049**	Cell cycle	1851	105	82.10	0.006
**Specific_1103P_gene**	**GO:0006629**	Lipid metabolic process	3920	92	76.98	0.041
	**GO:0007610**	Behavior	277	10	5.44	0.048
**Specific_1103P_promoter**	**GO:0007275**	Multicellular organism development	6389	160	147.97	0.0076

Supplementary Table 9. Go term enrichment analysis of genes of clusters in movement between scion and rootstock of CS/RGM combination

GO term enrichment of genes associated with clusters that have migrated from rootstock-to-scion (A) and those migrated from scion-to-rootstock (B).
CC: cellular component; MF: molecular function; BP: biological process

	Go term	Ontology	Description	Annotated	Significant	Expected	p-value
A	GO:0030677	CC	Ribonuclease P complex	1	1	0	0.0023
	GO:0005838	CC	Proteasome regulatory particle	7	1	0.02	0.0163
	GO:0005643	CC	Nuclear pore	13	1	0.03	0.0302
	GO:0043531	MF	ADP binding	826	20	2	4.6e-16
	GO:0005515	MF	Protein binding	4410	20	10.67	0.0024
	GO:0032977	MF	Membrane insertase activity	4	1	0.01	0.0096
	GO:0004097	MF	Catechol oxidase activity	7	1	0.02	0.0168
	GO:0017056	MF	Structural constituent of nuclear pore	9	1	0.02	0.0216
	GO:0004784	MF	Superoxide dismutase activity	10	1	0.02	0.0239
	GO:0031072	MF	Heat shock protein binding	10	1	0.02	0.0239
	GO:0007165	BP	Signal transduction	540	13	1.29	6.6e-11
	GO:0015743	BP	Malate transport	17	2	0.04	0.00074
	GO:0006801	BP	Superoxide metabolic process	16	1	0.04	0.03770
B	GO:0005886	CC	Plasma membrane	8508	87	61.94	0.0002
	GO:0005764	CC	Lysosome	338	6	2.46	0.0380
	GO:0005623	CC	Cell	27804	216	202.41	0.0413
	GO:0005515	MF	Protein binding	11350	161	100.92	1.5e-13
	GO:0030234	MF	Enzyme regulator activity	1151	18	10.23	0.016
	GO:0007165	BP	Signal transduction	5361	97	46.11	2.8e-13
	GO:0006950	BP	Response to stress	8565	113	73.67	4.6e-07
	GO:0006629	BP	Lipid metabolic process	3920	53	33.72	0.00064
	GO:0009987	BP	Cellular process	32387	292	278.56	0.00613

Supplementary Table 10. Characteristics of the clusters chosen as potential targets in the recipient compartement (scion CS of the heterograft combination).

The information of these 10 clusters in terms of genomic coordinates, ID, genomic annotation in gene bodies and 2 kb promoter regions and length are given in the first 5 columns. We then find the matches identified by blast on the CS genome

Coordinates of the clusters	Cluster_ID	Gene body	2 kb promoter region	cluster_length	Potential targetted areas on CS	length_blast	% cluster blast	pident_1	pident_final	mismatch
CM014908.1:1061180-1061218	Cluster_87	NA	NA	38	VITVvi_vCabSauv08_v1.1.hap1.chr05:1115840-1115878	38	100,00	97,37	97,37	1
CM014904.1:23780989-23781025	Cluster_19	genes	NA	36	VITVvi_vCabSauv08_v1.1.hap1.chr01:21606176-21606212	36	100,00	97,22	97,22	1
CM014906.1:16242012-16242130	Cluster_47	genes	promo	118	VITVvi_vCabSauv08_v1.1.hap1.chr03:10943001-10943119	118	100,00	95,76	95,76	5
CM014919.1:5207038-5207061	Cluster_476	genes	NA	23	VITVvi_vCabSauv08_v1.1.hap1.chr16:5194568-5194590	22	95,65	100,00	95,65	0
CM014910.1:19875438-19875633	Cluster_158	NA	promo	195	VITVvi_vCabSauv08_v1.1.hap1.chr15:19517299-19517387	88	45,13	95,45	43,08	4
CM014911.1:18899211-18899306	Cluster_204	NA	promo	95	VITVvi_vCabSauv08_v1.1.hap1.chr12:19873294-19873313	19	20,00	100,00	20,00	0
CM014914.1:13151822-13151949	Cluster_314	NA	NA	127	VITVvi_vCabSauv08_v1.1.hap1.chr11:6468431-6468558	127	100,00	96,85	96,85	4
CM014921.1:5180682-5180790	Cluster_548	NA	promo	108	VITVvi_vCabSauv08_v1.1.hap1.chr18:6914240-6914348	108	100,00	92,59	92,59	8
CM014919.1:22655943-22656048	Cluster_511	NA	NA	105	VITVvi_vCabSauv08_v1.1.hap1.chr16:21783546-21783645	99	94,29	96,97	91,43	3
CM014921.1:5166131-5166266	Cluster_547	NA	promo	135	VITVvi_vCabSauv08_v1.1.hap1.chr18:6914214-6914346	132	97,78	93,94	91,85	8

Supplementary Figure 1. Distribution of the small RNA clusters according to the number of reads per cluster
The number of reads per clusters was categorized into 7 classes for the clusters identified in the apexes and the roots of the homograft (CS/CS) and the heterograft (CS/RGM).

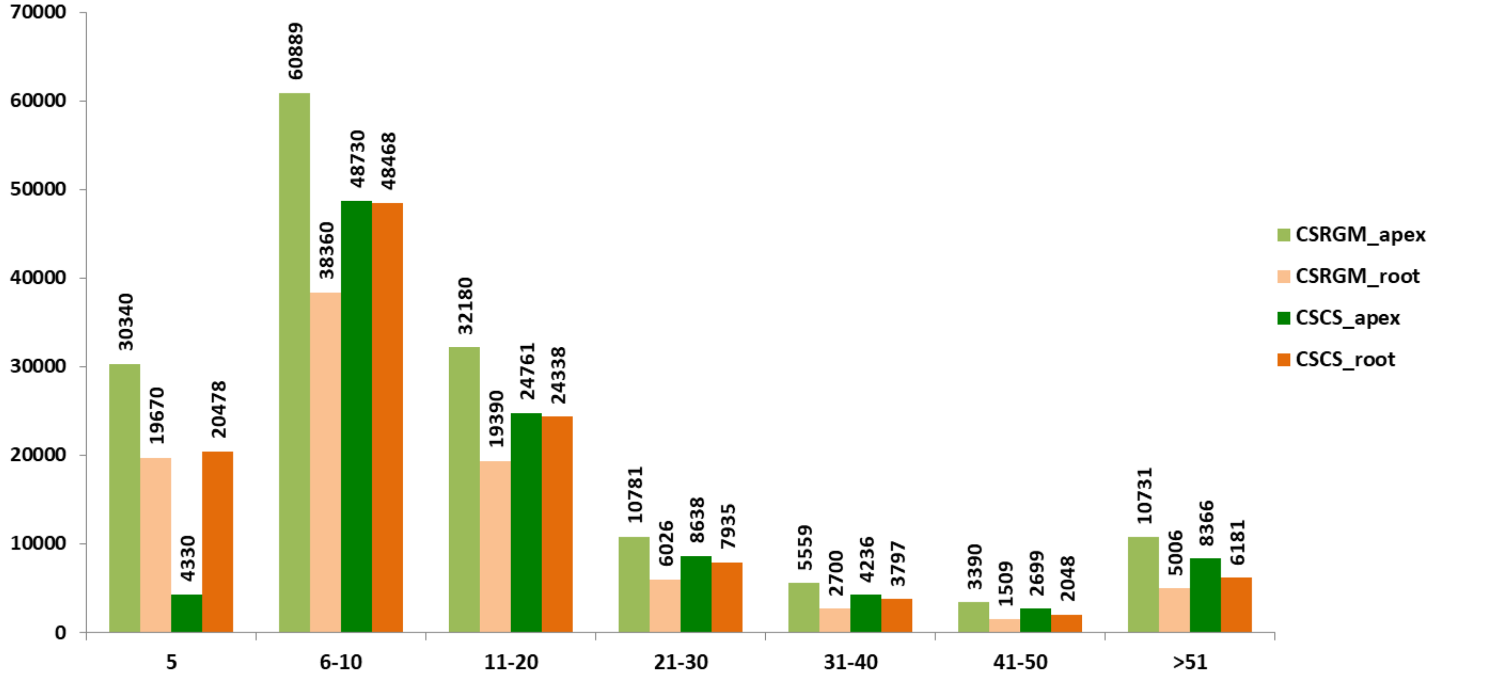

Supplementary Figure 2. Distribution of the miRNA clusters in apex and root compartiments of CS/CS and CS/RGM combinations
Proportion of miRNA clusters annotated as miRNA found ('known_miRNA') or not ('novel_miRNA') by blastn analysis of the 163 vvi-miRNA precursor sequences reported in miRbase 2.0 was represented in dark blue and skyblue, respectively.

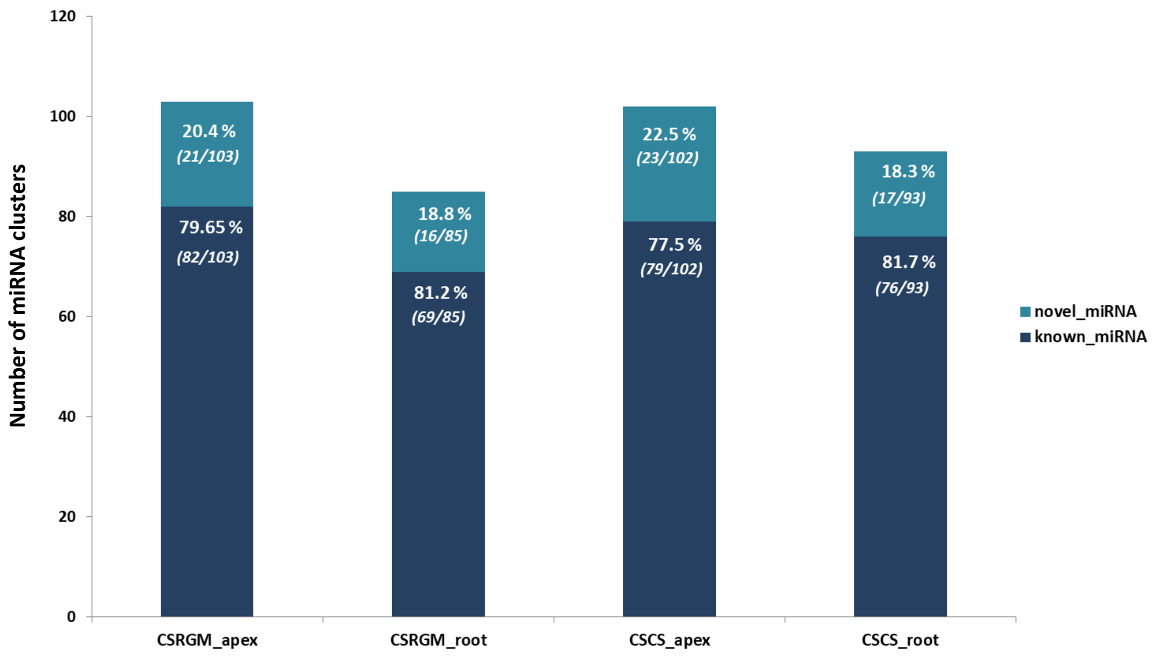

Supplementary Figure 3. Distribution of siRNA and miRNA clusters according to their sequence size

The distribution according to the sequence size from 20-nt to 24-nt was performed for siRNA (A) and miRNA (B) clusters in apexes and roots of the homograft (CS/CS) and the heterograft (CS/RGM) samples

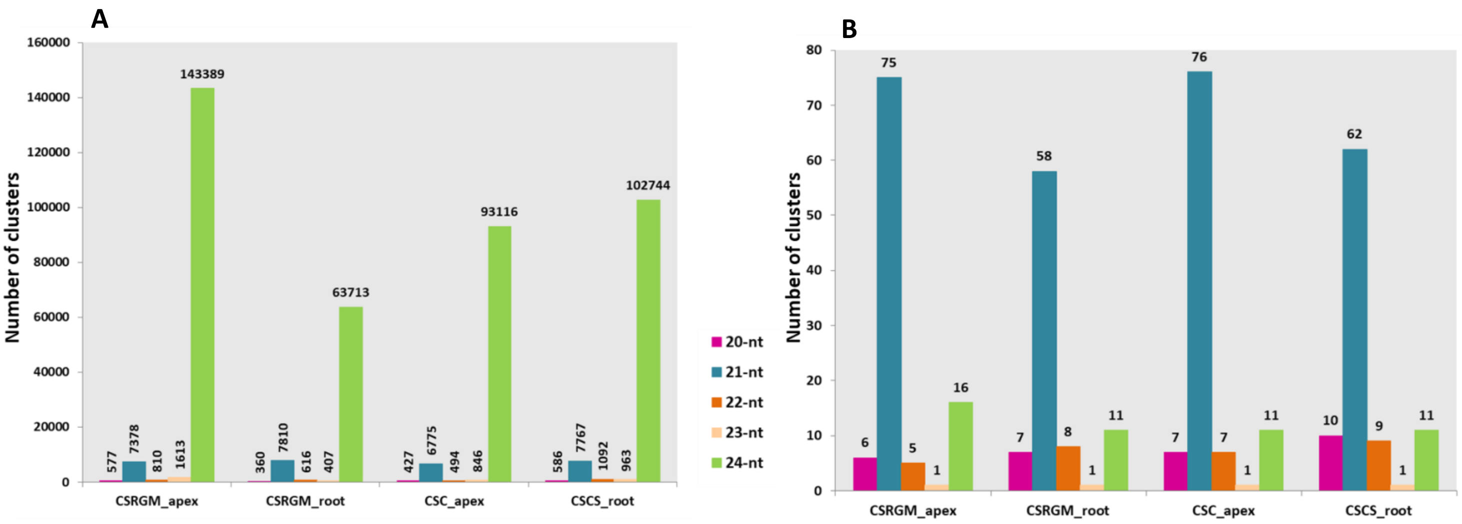

Supplementary Figure 4. Distribution of the small RNA clusters according to their annotation

Only clusters that have an annotation on gene and/or promotor and/or repeats are represented. In ‘gene’, ‘promoter’ and ‘repeats’ are referred to clusters annotated only in one of these three categories. We then distinguish the clusters annotated in at least two of these categories: ‘gene&promoter’, ‘gene&repeats’ and ‘promoter&repeats’. The percentages are calculated in relation to the number of total clusters which differs according to the plant compartment (leaves and roots) and the graft (homograft and heterograft).

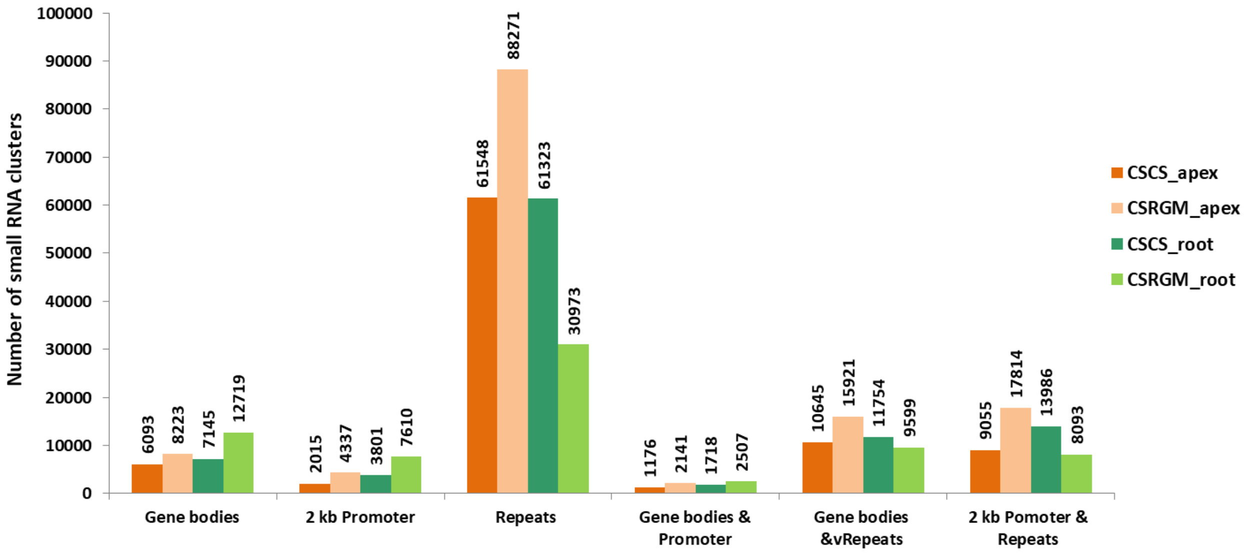

Supplementary Figure 5. Principal component analysis of the apexes replicates of the three graft combinations
The PCA was performed from the information of the number of reads per cluster found by Shorstack in each replicate of each graft combination. In red are the apexes samples of heterograft combinations CS/RGM and CS/1103P. In blue, the apexes samples of the homograft combination CS/CS.

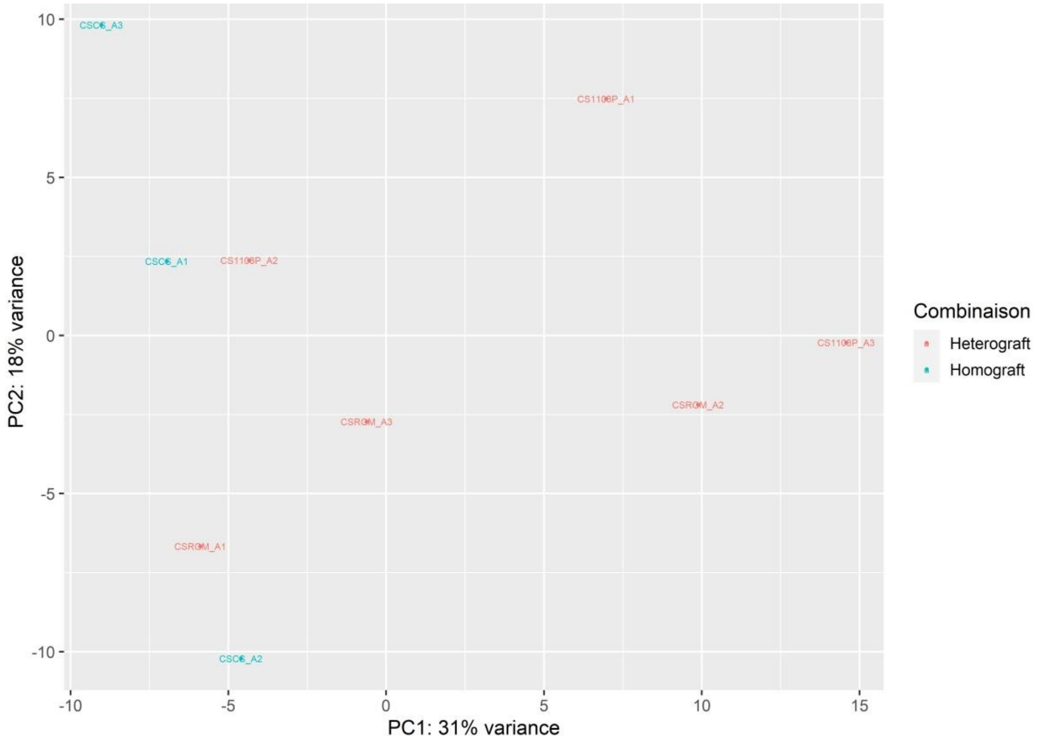

Supplementary Figure 6. Distribution of the smRNA clusters according to coefficient of variation categories for each graft combination
Coefficient of variation (CV in %) correspond to the variations in the number of reads per cluster between the replicates of each graft combination

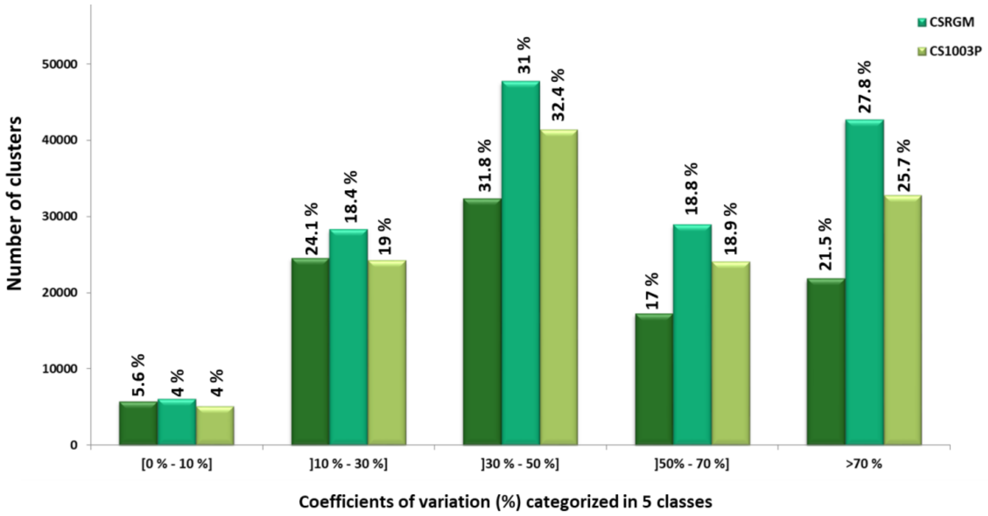

Supplementary Figure 7. Distribution of the smRNA clusters according to the number of reads per cluster
The number of reads per clusters were categorized into 7 classes for the clusters identified in the scion of the homograft (CS/CS) and both heterografts (CS/RGM & CS/1103P).

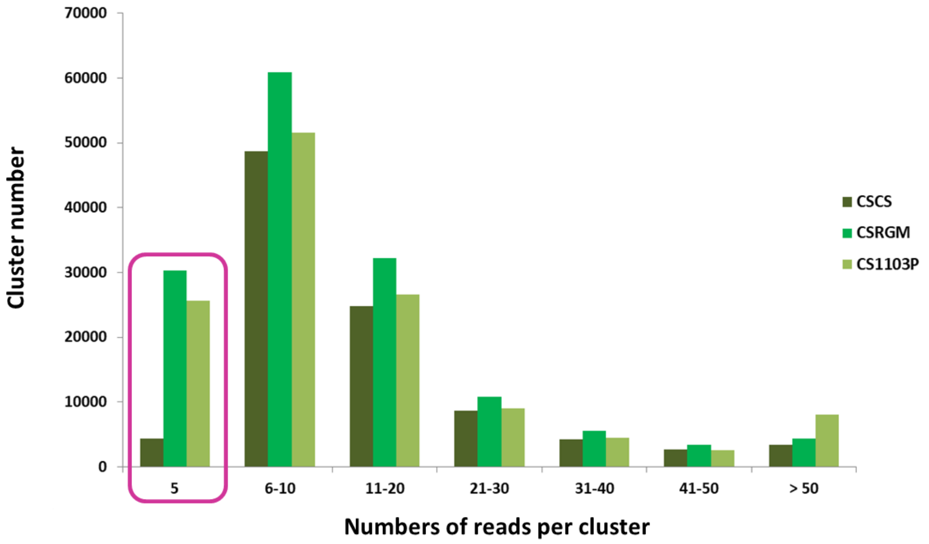

Supplementary Figure 8. Distribution of common clusters between CS/CS vs. CS/RGM and CS/CS vs. CS/1003P comparisons based on the differences in the number of reads of the clusters of each graft combination
The differences in the number of reads were categorized into 5 classes.

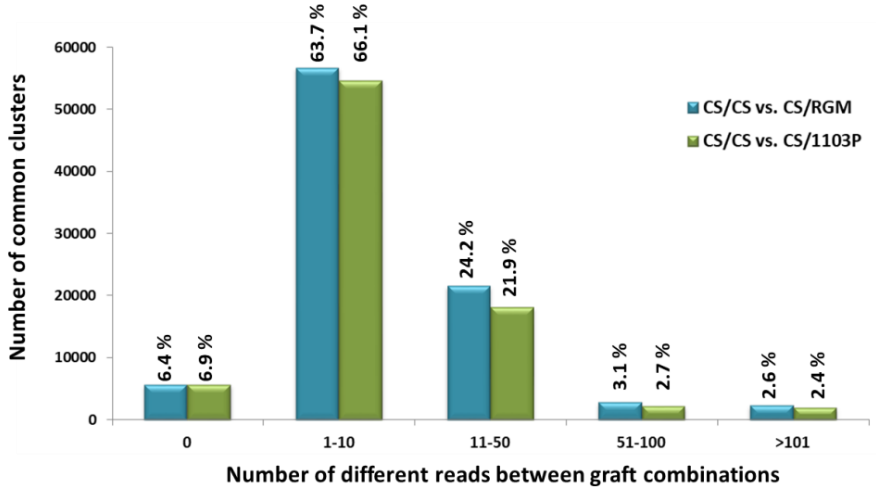

Supplementary Figure 9. A stepwise strategy to characterize mobile small RNAs from scion-to-rootstock and from rootstock-to-scion

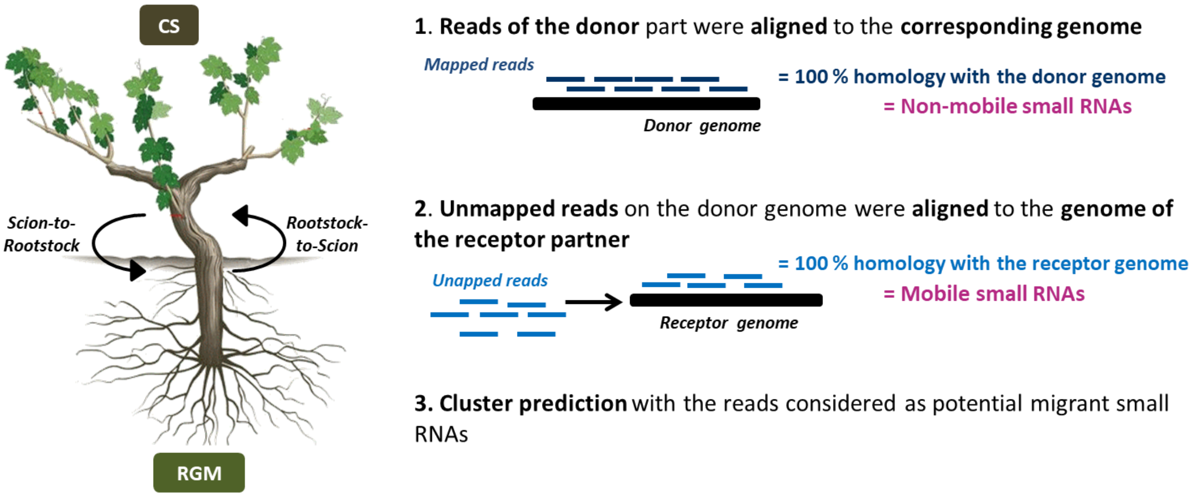

Supplementary Figure 10. Distribution of the mobile siRNA clusters from 20-nt to 24-nt according to their annotation (A) Rootstock-to-scion siRNA clusters (RT-SC) and (B) Scion-to-rootstock siRNA clusters (SC-RT)
Only clusters that have an annotation on gene and/or promotor and/or repeats are represented. In ‘gene’, ‘promoter’ and ‘repeats’ are referred to clusters annotated only in one of these three categories. The numbers above the bars correspond to the percentages of each category calculated on the total number of mobile siRNAs clusters identified (i.e 228 RT-SC and 2725 SC-RT).

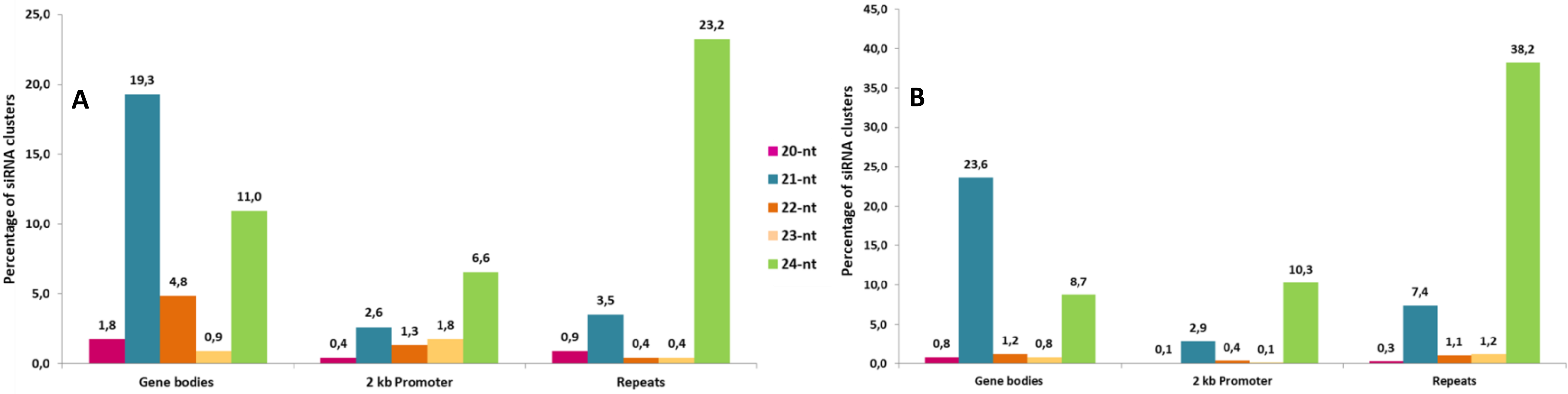

Supplement: Web_Material_uhab067 [file web_material_uhab067.zip › supplementary information.pdf]
